# Inhibition of PAI‐1 limits chemotherapy resistance in lung cancer through suppressing myofibroblast characteristics of cancer‐associated fibroblasts

**DOI:** 10.1111/jcmm.14205

**Published:** 2019-02-07

**Authors:** Takeshi Masuda, Taku Nakashima, Masashi Namba, Kakuhiro Yamaguchi, Shinjiro Sakamoto, Yasushi Horimasu, Shintaro Miyamoto, Hiroshi Iwamoto, Kazunori Fujitaka, Yoshihiro Miyata, Hironobu Hamada, Morihito Okada, Noboru Hattori

**Affiliations:** ^1^ Department of Respiratory Internal Medicine Hiroshima University Hospital Hiroshima Japan; ^2^ Department of Surgical Oncology Research Institute for Radiation Biology and Medicine Hiroshima University Hiroshima Japan

**Keywords:** cancer‐associated fibroblast, chemotherapy, lung cancer, myofibroblast, plasminogen activator inhibitor‐1

## Abstract

Plasminogen activator inhibitor‐1 (PAI‐1) promotes pulmonary fibrosis through increasing myofibroblast (MF) characteristics, expressing alpha‐smooth muscle actin (α‐SMA) in fibroblasts. Fibroblasts in the tumour stroma are called cancer‐associated fibroblasts (CAFs). Some CAFs have MF characteristics and substantially promote tumour progression and chemotherapy resistance. This study determined whether inhibition of PAI‐1 suppressed MF characteristics of CAFs and limited chemotherapy resistance in lung cancer. To investigate cellular PAI‐1 expression and its correlation with α‐SMA expression of CAFs, 34 patients’ paraffin‐embedded lung adenocarcinoma tissue sections were immunohistochemically stained for PAI‐1 and α‐SMA. Immunohistochemical analysis of lung adenocarcinoma tissues showed that PAI‐1 expression was correlated with that of α‐SMA (*r* = 0.71, *p* < 0.001). Furthermore, in vitro, α‐SMA expression of CAFs was limited by PAI‐1 inhibition, and apoptosis of CAFs was increased. In addition, the effectiveness of cisplatin on lung cancer cells co‐cultured with CAFs was increased by suppressing α‐SMA expression using PAI‐1 inhibitor. In lung adenocarcinoma tissues, PAI‐1 expression was associated with T factor and TNM stage. Our data suggest that inhibition of PAI‐1 increased the chemotherapeutic effect on lung cancer through suppressing the MF characteristics of CAFs. Hence, PAI‐1 might be a promising therapeutic target for patients with chemotherapeutic‐resistant lung cancer with CAFs.

## 1 INTRODUCTION

Lung cancer is reported to be a highly progressive disease and frequently resistant to chemotherapy, thus the patients with advanced lung cancer have poor prognosis.^1^ Nowadays, the immune therapy and molecular target therapy showed promising anti‐tumour efficacy for advanced non‐small cell lung cancer (NSCLC), although their prognosis is still not satisfactory.^2^, ^3^ Therefore, novel treatment strategy is urgently needed.

Fibroblasts in the tumour stroma, called cancer‐associated fibroblasts (CAFs), have been reported to induce chemotherapeutic resistance in lung cancer. Actually, a large number of studies showed that CAFs promoted the metastatic ability, invasion and proliferation of lung cancer cells, which induce resistance to chemotherapy by secreting a number of growth factors and inflammatory chemokines.^4^, ^5^ Furthermore, a part of CAFs has myofibroblast (MF) characteristic with the expression of alpha‐smooth muscle actin (α‐SMA), which substantially promotes chemotherapeutic resistance by expressing high levels of inflammatory factors and chemokines.^6^, ^7^ Thus, the suppression of the MF characteristics of CAFs can be a novel treatment strategy for a chemotherapeutic‐resistant lung cancer with myofibroblastic CAFs.

Plasminogen activator inhibitor‐1 (PAI‐1), which is produced by endothelial cells of blood vessels, inflammatory cells and fibroblasts, is a glycoprotein with 47‐kDa molecular size and inhibits the activation of plasminogen activator.^8^ PAI‐1 was reported to promote pulmonary fibrosis by blocking fibrinolysis in the lung parenchyma,^9^ and also its association with TGF‐β induces the differentiation of fibroblasts into MFs.^10^ PAI‐1 has been also reported to be involved in resistance to chemotherapy and tumourigenesis in various types of cancers.^11^, ^12^, ^13^ In vivo studies revealed the significance of PAI‐1 in regulating tumour angiogenesis.^14^, ^15^, ^16^ Furthermore, several in vitro studies have shown that PAI‐1 has a direct pro‐proliferative^17^ and anti‐apoptotic^18^ effect on the cancer cells.

From these observations, we suggested that the inhibition of PAI‐1 could increase the efficacy of chemotherapy on lung cancer by suppressing the MF characteristics of CAFs. In this study, we aimed to elucidate this hypothesis using PAI‐1 inhibitor.

## 2 MATERIALS AND METHODS

### 2.1 Materials

Murine Lewis lung carcinoma cells (LLC) and mouse lung fibroblasts (MLFs) were obtained from and authenticated by American Type Culture Collection (Manassas, VA, USA). Human lung adenocarcinoma epithelial cells (A549) and *epidermal growth factor receptor* (*EGFR)* gene mutant lung cancer cell lines PC‐9 were provided by RIKEN BRC through the National Bio‐Resource Project of the MEXT/AMED, Japan. Human lung fibroblast cells (MRC‐5) were purchased from the Japanese Collection of Research Bioresources Cell Bank (Tokyo, Japan). Original human fibroblasts were obtained from carcinomatous pleural effusions in patients with lung adenocarcinoma. We called these fibroblasts CAF, because these fibroblasts existed with cancer cells in the pleural effusion, similar to fibroblasts in the tumour stroma. Briefly, the method to purify these fibroblasts was as follows. The cells in the pleural effusion were collected by centrifugation at 1500 r.p.m for 3 minutes. After that, the cells were resuspended with 20 mL of Roswell Park Memorial Institute (RPMI) medium. To divide the tumour cells and fibroblasts from other cells, the cells were centrifuged at 1000 r.p.m for 10 seconds. This final procedure was repeated three times. In the passage process, only cells with spindle shape survived. To confirm that these cells were fibroblasts, we investigated the mRNA expression of fibroblast activation protein (FAP), which is a specific marker of fibroblasts, and α‐SMA by quantitative real‐time PCR (qPCR). Thirty‐four invasive lung adenocarcinoma tissues samples were obtained from the patients who had undergone surgery at our hospital from January 2001 through December 2011.

International Cancer Control TNM Classification of Malignant Tumours 7th edition^19^ was used for the classification of variables. This study was approved by the Hiroshima University Institutional Review Board (No. E136‐1) and conducted in accordance with the ethical standards established by the Helsinki Declaration of 1975. To obtain consent of the patients, opt‐out method was applied in this retrospective study.

### 2.2 Cell culture and treatment

A549, PC‐9 and LLC cells were cultured in RPMI supplemented with 10% foetal bovine serum (FBS) and 1% penicillin‐streptomycin. MRC‐5 cells, MLFs and CAFs were cultured in EMEM. These cells were incubated at 37°C in a 5% CO_2_ incubator and used within 6 months after resuscitation. MRC‐5 cells, MLFs and CAFs were seeded at a density of 1 × 10^5^ cells/well in six‐well plates for qPCR, ELISA, quantitative proteomic analysis and phospho‐kinase array. In addition, these fibroblasts were seeded at a density of 1.5 × 10^4^ cells/insert well in 24‐well plates for chemotherapy effect, in the 24‐well plates for proliferation and cell cycle assays. For apoptosis assay, these cells were seeded at a density of 1 × 10^4^ cells/well in 96‐well plates. After the plating, these fibroblasts were cultured in EMEM supplemented with 10% FBS for 12 hours.

Thereafter, MLFs and MRC‐5 cells were pre‐incubated with or without SK‐216, a PAI‐1 inhibitor, (20 or 50 μmol/L) in a serum‐free medium for 1 hour followed by stimulation with TGF‐β1 (mouse or human recombinant TGF‐β1, 5 ng/mL, R&D Systems, Minneapolis, MN). On the other hand, CAFs were cultured with or without SK‐216 (100, 250, or 500 μmol/L) in the medium with FBS. These cells were used for various analyses, 36 hours after the treatment.

### 2.3 Reagents

SK‐216 (Figure S1) was chemically synthesized and supplied by Shizuoka Coffein Co., Ltd. (Shizuoka, Japan). The IC_50_ for SK‐216 was determined to be 44 μmol/L as reported in international patent WO04/010996. Afatinib and cisplatin were purchased from Wako Junyaku Kogyo Co. (Osaka, Japan).

### 2.4 Quantitative real‐time PCR

Total RNA was isolated with RNeasy Mini Kits (Qiagen, Valencia, CA, USA). The isolated total RNA was reverse transcribed into cDNA using a High Capacity RNA‐to‐cDNA^™^ Kit (Applied Biosystems, Framingham, MA, USA) following the manufacturer's instructions. Quantitative RT‐PCR was performed with an ABI Prism 7700 (Applied Biosystems). mRNA expression levels were evaluated and normalized to β‐actin as an endogenous reference. Primers used were as follows: E‐cadherin (TaqMan Gene Expression Assay ID Hs01023894_m1, Applied Biosystems); fibronectin (Hs01549976_m1); α‐SMA (Hs00426835_g1, Mm00725412_s1); FAP (Hs00990791_m1); and β‐actin (4352935E, 4352341E).

### 2.5 Verification of knockdown effect of PAI‐1‐siRNA in vitro

Fibroblasts were transfected at 70% confluence for 36 h with 10 nM Silencer Select small interfering RNA (siRNA) targeting human PAI‐1 (PAI‐1‐siRNA) or a Silencer Select negative control siRNA (NC‐siRNA) (Thermo Fisher Scientific, Waltham, MA, USA). The target sequences of siRNA against PAI‐1‐siRNA were as follows: sense, 5′‐ GACCAACAAGUUCAACUAUtt ‐3′ and antisense, 5′‐ AUAGUUGAACUUGUUGGUCtg‐ ‐3′. The Lipofectamine RNAiMAX transfection reagent (Invitrogen, Carlsbad, CA, USA) was used for transfection in each group. The cells and cell culture supernatant were collected 36 hours after transfection.

### 2.6 Quantification of PAI‐1 and TGF‐β protein

Total PAI‐1 or TGF‐β secreted into serum‐free culture medium for 36 hours and in the serum were measured using an ELISA kit (R&D systems, Minneapolis, MN) following the manufacturer's instructions.

### 2.7 Immunohistochemical staining

Human tumour sections were incubated with a PAI‐1 (Santa Cruz, CA, USA), α‐SMA (Abcam, Cambridge, UK) rabbit polyclonal antibody and followed by 30 minutes of incubation with a biotinylated goat anti‐rabbit IgG antibody (Vector Laboratories, Burlingame, CA, USA). The immunoreaction was amplified with a Vectastain ABC kit (Vector Laboratories). Isotype control antibody (DaKo, Santa Clara CA, USA) was used as a negative control. In addition, immunohistochemical analysis for CD3 and CD79a was conducted with Ventana Benchmark automated staining system (Ventana Medical Systems, Tucson, AZ, USA) using Ventana reagents. To detect CD3 or CD79a, CD3 and CD79a antibodies (Roche Molecular Systems, Pleasanton, CA, USA) were used. All immunoreactions were visualized by incubation with a 3, 3‐diaminobenzidine solution acting as a chromogen. The sections were then counterstained with haematoxylin and dehydrated. Immunofluorescence staining for the detection of α‐SMA was performed following our previous report.^10^ To detect α‐SMA, α‐SMA antibodies (R&D systems) were used. Nuclei were stained with 4’, 6‐diamidino‐2‐phenylindole (Vector Laboratories). All images were captured using a microscope at a magnification of 40 or 200× (model BZ‐9000; Keyence, Osaka, Japan). The stromal area, excluding cancer cells, was manually identified and calculated using Dynamic cell count software BZ‐HIC (Keyence). Afterwards, the PAI‐1 or α‐SMA positive area was measured using the software. Finally, the percentage of PAI‐1 or α‐SMA positive area in the stromal area was determined.

### 2.8 iTRAQ proteomics

A commercial iTRAQ analysis system (Filgen, Nagoya, Japan) was used with mass spectrometry. Briefly, the total protein from whole cells was purified, and 100 μg of protein samples was reduced and alkylated prior to trypsin digestion, and the resulting peptides were lyophilized and reconstituted before labelling with sham control‐iTRAQ 113 and TAC 8W‐iTRAQ 115 according to the manufacturer's instructions (AB SCIEX). Labelled digests were combined into sample mixtures, and protein identification and relative iTRAQ quantitation were performed with an AB SCIEX TripleTOF 5600 mass spectrometer with ProteinPilot™ software version 4.5 using the Paragon™ Algorithm 4.5.0.0.

### 2.9 Phospho‐mitogen‐activated protein kinase (MAPK) array

The total protein from whole cells were purified. Cell lysates were incubated with the Human Phospho‐MAPK Array Kit (R&D Systems) according to the manufacturer's instructions. Phospho‐MAPK Array data was visualized using a chemiluminescence detection system (WSE‐6100; ATTO, Tokyo, Japan) and measured using ImageSaver6 (ATTO).

### 2.10 Apoptosis and cell cycle assay

Apoptosis was investigated using ApoLive‐Glo Multiplex Assay (Promega, WI, USA) and ApoStarand ELISA Apoptosis Detection Kit (Enzo Life Sciences, Farmingdale, USA) following the manufacturer's instructions. Cell cycle assay was performed with Cell‐Clock™ Cell Cycle Assay kit (Biocolor Life Science Assays, Carrickfergus, UK).

### 2.11 Proliferation assay

The proliferation of cells was analysed using the cell counting kit‐8 (DOJINDO, Kumamoto, Japan) following the manufacturer's instructions.

### 2.12 In vitro analysis of efficacy of chemotherapy drugs on cancer cells co‐cultured with fibroblasts

As described in the Cell culture and treatment section, after 36 hours, the fibroblasts were treated with SK‐216 and TGF‐β1, the insert well with the fibroblasts was transferred into a new 24‐well plate. Thereafter, A549, PC‐9 or LLC cells were seeded at a density of 4 × 10^4^ cells/well in the 24‐well plate, and co‐culture of cancer cells with fibroblast was started. Figure S2 shows the scheme of this in vitro model. After 36 hours, cisplatin (10 μmol/L) or afatinib (1 μmol/L) was added to the medium. The proliferation of cancer cells was determined after 36 hours.

### 2.13 Statistical analysis

Statistical analyses were undertaken using SPSS 17 (SPSS Japan). All the results are expressed as mean ± standard deviation, and the Student *t* test or Mann‐Whitney *U* test was used to evaluate statistical differences between the groups. Correlations were analysed with Pearson's correlation coefficient test. A *P *> 0.05 was considered statistically significant.

## 3 RESULTS

### 3.1 The level of PAI‐1 was correlated with the proportion of MF characteristics of CAFs

**Figure 1:**
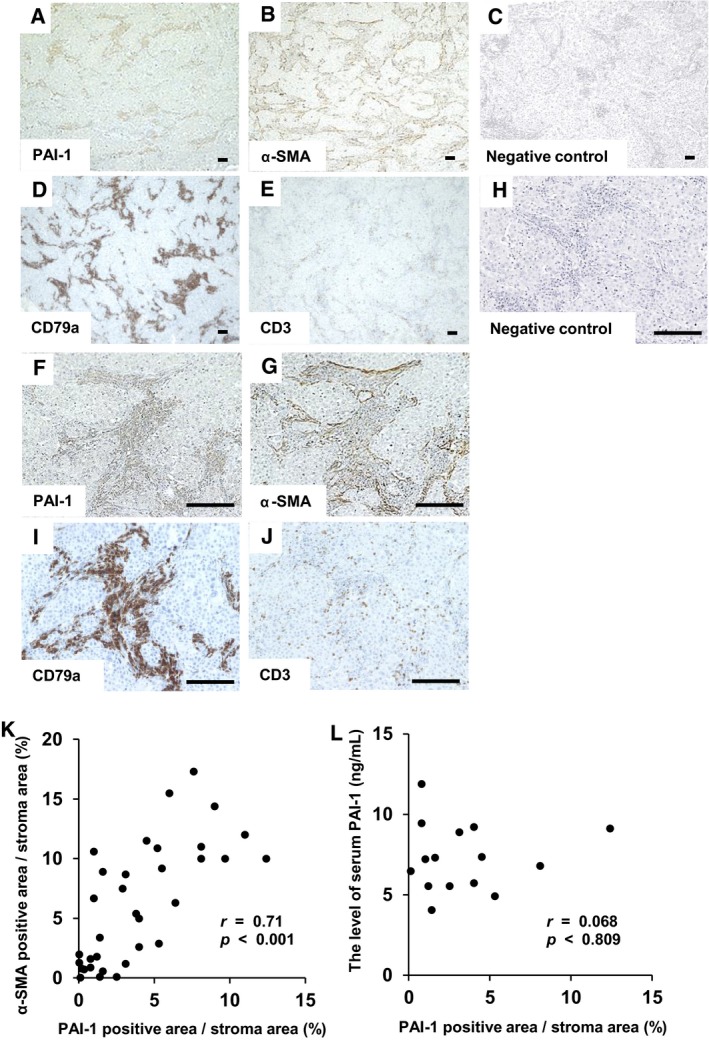
Immunohistochemical analysis of lung adenocarcinoma tissue sections and correlation of plasminogen activator inhibitor‐1 (PAI‐1) and alpha‐smooth muscle actin (α‐SMA) expression. Representative immunohistochemical staining of (A) PAI‐1, (B) α‐SMA, (C) negative control for PAI‐1 and α‐SMA, (D) CD79a and (E) CD3 in lung adenocarcinoma tissue sections. (A)‐(E), magnification: ×40. (F)‐(J), magnification: ×200. Scale bar = 50 μm. The proportion of PAI‐1 or α‐SMA positive area/stroma was calculated as described in Materials and Methods. (K) The correlation between the positive area of PAI‐1 and α‐SMA in the tumour stroma (*r* = 0.71, *P* < 0.001). (L) The correlation between the positive area of PAI‐1 in the stroma and the concentration of PAI‐1 in the serum (*r* = 0.068, *P* = 0.810). The data were analysed with the Pearson's correlation coefficient test

Because α‐SMA is considered as a specific marker of the MF characteristics of fibroblast,^20^ we investigated α‐SMA expression of fibroblasts in this study. To investigate cellular PAI‐1 expression and its correlation with α‐SMA expression of CAFs in lung adenocarcinoma, 34 patients’ lung adenocarcinoma tissue sections were immunohistochemically stained with antibodies specific for PAI‐1 and α‐SMA. The patients’ characteristics are shown in Table 1. Immunohistochemical analysis revealed that PAI‐1 expression was higher in the tumour stroma compared with cancer cells (Figure 1A and F). The morphology of cells in the tumour stromal area was characteristic of fibroblasts or lymphocytes. Therefore, tissue sections were immunohistochemically stained for α‐SMA, CD79a and CD3. The staining showed that the cells in tumour stroma were α‐SMA positive fibroblasts (Figure 1B and G), CD79a positive B lymphocytes (Figure 1D and I) and CD3 positive T lymphocytes (Figure 1E and J). Furthermore, immunohistochemical analysis of lung adenocarcinoma tissues showed that the PAI‐1 level was correlated with that of MF with α‐SMA expression (*r* = 0.71, *P* < 0.001) (Figure 1K). On the other hand, this PAI‐1 expression level was not significantly correlated with that in the serum (Figure 1L).

**Table 1:** Patient characteristics

Patient characteristics	n = 34
Age (years)	
Median (range)	64 (56‐84)
Sex	
Male/female	27/7
ECOG PS	
0−1/≥2	32/2
Smoking amount, pack‐years	
<40/≥40	9/25
T factor	
1/2/3/4	12/16/2/4
N factor	
0/1/2/3	20/8/5/1
Stage	
I/II/III	16/8/10

ECOG PS, Eastern Cooperative Oncology Group performance status.

### 3.2 PAI‐1 was associated with the maintenance of MF characteristics of fibroblasts

**Figure 2:**
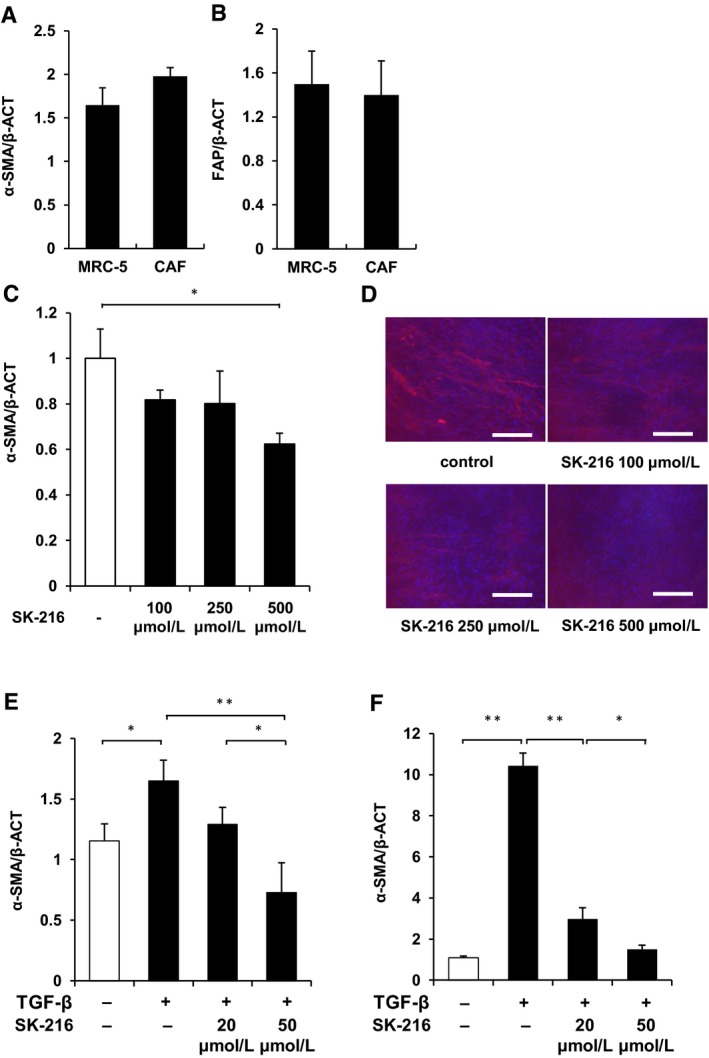
Plasminogen activator inhibitor‐1 (PAI‐1) was associated with maintaining myofibroblast (MF) characteristics of fibroblasts. Cancer‐associated fibroblasts (CAFs) or MRC‐5 cells were cultured for 36 hours, these cells were harvested, and total RNA was extracted. The mRNA expression of (A) α‐SMA and (B) fibroblast activation protein were quantified using real‐time quantitative RT‐PCR. Results are expressed as fold change from the control and reflect the mean ± SD. CAFs were cultured with or without SK‐216 (100, 250, or 500 μmol/L) in the medium. Mouse lung fibroblasts (MLF) or MRC‐5 cells were cultured for 12 hours and these cells were pre‐incubated with or without SK‐216 (20 or 50 μmol/L) in the medium for 1 hour followed by stimulation with TGF‐β1. After incubation for 36 hours, CAF, MLF and MRC‐5 cells were harvested and total RNA was extracted. The mRNA expression of α‐SMA was quantified using quantitative RT‐PCR. mRNA expression of α‐SMA in the (C) CAF, (E) MLF and (F) MRC‐5 cells. (D) CAFs stained for α‐SMA (red) and nuclei (blue). Magnification: ×200. Scale bar = 50 μm. The data represent the mean values of three samples (±SD) and were analysed with the student *t* test. *P < 0.05; **P < 0.01

First, to confirm that the cells with spindle shape obtained from the carcinomatous pleural effusion were fibroblasts, we investigated the mRNA expression of α‐SMA and FAP by qPCR. qPCR revealed that the cells expressed these mRNA similar to MRC‐5 cells (Figure 2A, B). Therefore, we confirmed these cells were CAFs. Next, to examine whether PAI‐1 had an association with maintaining the MF characteristics of CAFs, we investigated whether PAI‐1 inhibition limited the α‐SMA expression of CAFs. As Figure 2C and D showed, the α‐SMA expression of CAFs was limited by SK‐216, a specific PAI‐1 inhibitor in dose‐dependent manner. This result showed that PAI‐1 was involved in maintaining the MF characteristics of CAFs. The main progenitors of CAFs seem to be the local fibroblasts and these fibroblasts obtain MF characteristics by TGF‐β stimulation in the tumour stroma.^21^, ^22^ Therefore, to investigate whether PAI‐1 had association with normal fibroblasts obtaining MF characteristics in the tumour microenvironment, we examined whether the α‐SMA expression of MLF and MRC‐5 cell lines after TGF‐β stimulation was limited by PAI‐1 inhibition. As shown in Figure 2E and F, PAI‐1 inhibition limited the expression of α‐SMA. These results indicated that PAI‐1 was associated with normal fibroblasts obtaining the MF characteristics after TGF‐β stimulation.

The difference in the concentrations of PAI‐1 inhibitor required to limit α‐SMA expression between CAFs, MRC‐5 cells and MLFs was observed. The concentration of PAI‐1 secreted by CAFs in the culture medium was not significantly different from that of MRC‐5 cells (Figure S3A). On the other hand, CAFs were cultured in the medium with FBS, but MRC‐5 and MLF cells were cultured in the serum‐free medium. Therefore, the discrepancy was likely due to several growth factors in the FBS that promoted α‐SMA expression.

Next, we performed siRNA knockdown experiments to confirm the association of PAI‐1 and MF characteristics of fibroblasts. PAI‐1 siRNA suppressed the PAI‐1 mRNA expression level of CAFs to 10% of NC‐siRNA (Figure S2B). PAI‐1 concentrations in the medium were also shown to be significantly reduced by using PAI‐1‐siRNA compared with NC‐siRNA (Figure S2C). In addition, the α‐SMA expression of CAFs was significantly suppressed by the siRNA (Figure S2D). In the experiments using MRC‐5 cells, similar results were observed (Figure S2E‐G).

### 3.3 Analysis of the underlying the association between PAI‐1 and MF phenotype of CAFs

**Figure 3:**
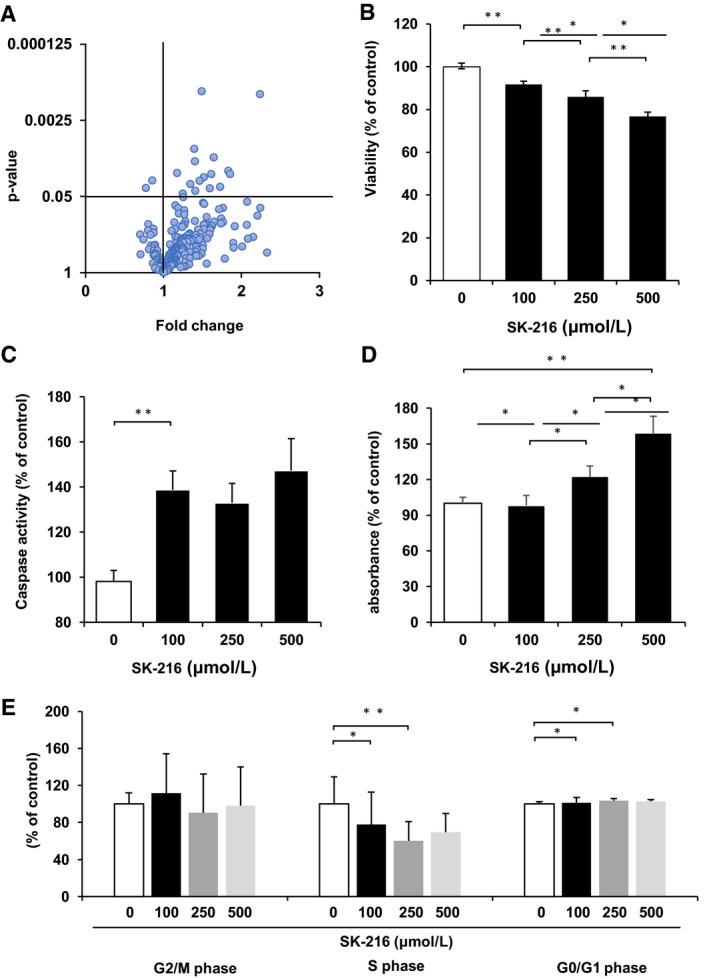
Plasminogen activator inhibitor‐1 (PAI‐1) maintained MF characteristics of cancer‐associated fibroblasts (CAFs) through inhibiting apoptosis. CAFs were cultured with or without SK‐216 (500 μmol/L) in the medium. After 36 hours, the cell lysates were subjected to quantitative proteomic analysis. (A) Volcano plot showing differential expression of proteins. CAFs were cultured with or without SK‐216 (100, 250, or 500 μmol/L) in the medium 36 hours. (B) Cell viability, (C) caspase activity, (D) single strand DNA expression level and (E) cell cycle analysis in the CAFs treated by SK‐216 and untreated control were examined as described in Materials and Methods. The data of (B‐E) represent the mean values of three samples (±SD) and were analysed with the student *t* test. *P < 0.05; **P < 0.01

To investigate the mechanism by which PAI‐1 maintained the MF characteristics of the CAFs, we performed quantitative proteomic analysis comparing the CAFs treated by SK‐216 with untreated control CAFs using the iTRAQ analysis system. The level of 468 proteins in the CAFs treated by SK‐216 was changed when compared with the untreated control. Of the 468 proteins, the expression levels of 16 proteins were significantly up‐regulated (Figure 3A). In addition, of the 16 proteins, 10 proteins are related to apoptosis. The names of the 16 proteins are shown in Table S1.

### 3.4 PAI‐1 maintained the MF characteristics of CAFs by inhibiting apoptosis

The iTRAQ analysis revealed that the expression level of many apoptosis‐related proteins was elevated in the CAFs by PAI‐1 inhibition. Thus, we investigated whether PAI‐1 maintained the MF characteristics by inhibiting apoptosis of CAFs. We showed that PAI‐1 inhibition in CAFs by treatment with SK‐216 significantly decreased cell viability (Figure 3B) and increased the caspase activity and the amount of single strand DNA (Figure 3C and D). In addition, cell cycle analysis revealed the proportion of S phase cells decreased and G0/G1 phase cells significantly increased in the SK‐216‐treated groups than in the control. However, the degree of phosphorylation of MAPK protein was not different between CAFs treated with SK‐216 and untreated control (Figure S4A and B).

### 3.5 Inhibition of PAI‐1 increased the effect of chemotherapy on lung cancer cells co‐cultured with fibroblasts through suppressing the MF characteristics of fibroblasts

**Figure 4:**
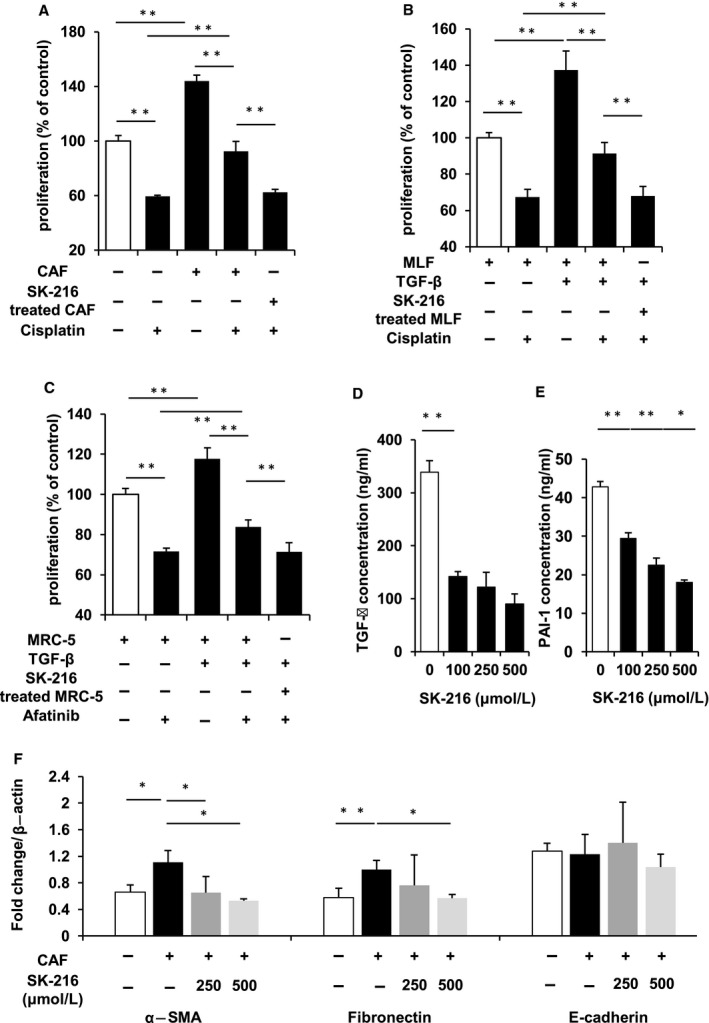
Inhibition of plasminogen activator inhibitor‐1 (PAI‐1) increased the effect of chemotherapy on lung cancer cells co‐cultured with fibroblasts through suppressing the MF characteristics of fibroblasts. (A) Cancer‐associated fibroblasts (CAFs) were cultured with or without SK‐216 (500 μmol/L) in the insert well on 24‐well plates for 36 hours. The insert well with CAFs was transferred into a new 24‐well plate. A549 cells were seeded at a density of 4 × 10^4^ cells/well in the 24‐well plate. After 12 hours, cisplatin (10 μmol/L) was added to the medium. Thereafter, the proliferation of cancer cells was determined after 36 hours. (B) MRC‐5 (C) MLF were pre‐incubated with or without SK‐216 (50 μmol/L) in the medium for 1 hour followed by stimulation with TGF‐β1 in the insert well on 24‐well plates. After incubation for 36 hours, the insert well with the MRC‐5 or MLF was transferred into a new 24‐well plate. Thereafter, (B) PC‐9 or (C) LLC cells were seeded at a density of 4 × 10^4^ cells/well in the 24‐well plate. After 12 hours, (B) cisplatin (10 μmol/L) (C) afatinib (1 μmol/L) were added to the medium. Thereafter, the proliferation of cancer cells was determined after 36 hours. (D), (E) CAFs were cultured with or without SK‐216 (100, 250, or 500 μmol/L) in the medium. ELISA assay of PAI‐1 and TGF‐β level secreted by CAFs in the medium. (F) A549 were co‐cultured with CAFs with or without SK‐216 (100, 250, or 500 μmol/L) in the medium for 36 hours. The cells were harvested, and total RNA was extracted. The expression of α‐SMA and fibronectin as mesenchymal marker, e‐cadherin as epithelial marker was examined by quantitative RT‐PCR. The data represent the mean values of four or six samples (±SD) and were analysed with the student *t* test. *P < 0.05; **P < 0.01

We investigated whether the suppression of the MF characteristics of CAFs by PAI‐1 inhibition increased the effect of chemotherapy (Figure S2). As shown in the Figure 4A, the proliferation of A549 cells co‐cultured with CAFs was significantly increased than that of A549 cells cultured without CAFs. In addition, the effect of cisplatin on A549 cells co‐cultured with CAFs was significantly decreased compared to the effect on cells cultured without CAFs. On the other hand, when CAFs were treated with SK‐216, the effect of cisplatin was significantly increased. These results suggest that the inhibition of PAI‐1 limits the resistance to chemotherapy via suppressing the MF characteristics of CAFs. Further, we investigated whether PAI‐1 inhibition increased the effect of chemotherapy through suppressing the MF characteristics of normal fibroblasts after TGF‐β stimulation. The effect of cisplatin on LLC cells co‐cultured with the TGF‐β‐stimulated MLFs was significantly decreased compared to the effect on cells without TGF‐β stimulation. On the other hand, the chemotherapeutic efficacy was increased when the MF characteristics of MLFs were suppressed by SK‐216 (Figure 4B). In addition, we examined whether the inhibition of PAI‐1 increased the effect of a molecular therapeutic drug through suppressing the MF characteristics. We showed the effect of afatinib, tyrosine kinase inhibitor for lung cancer with *EGFR* gene mutation, on PC‐9 cells similarly increased through suppressing the MF characteristics of MRC‐5 by SK‐216 (Figure 4C). These results indicated that the inhibition of PAI‐1 limited the resistance to cytotoxic and molecular chemotherapy drugs through suppressing the MF characteristics of normal fibroblasts after TGF‐β stimulation. In addition, to investigate the mechanism by which PAI‐1 inhibition limited the resistance to chemotherapy in cancer cells, we examined the concentration of PAI‐1 and TGF‐β secreted in the medium. ELISA assay revealed that these proteins levels were significantly higher in untreated CAFs when compared to those treated with SK‐216 (Figure 4D and E). This result suggested that PAI‐1 and TGF‐β secreted by CAFs could influence the effect of chemotherapy. TGF‐β is reported to be a strong inducer of epithelial mesenchymal transition (EMT),^23^ and we showed previously that PAI‐1 had association with EMT as a downstream effector of TGF‐β.^10^, ^24^ Therefore, we investigated the level of the EMT marker in the A549 cells co‐cultured with CAF treated with SK‐216 and untreated control. qPCR showed the expression of mesenchymal markers, α‐SMA and fibronectin was significantly higher in control than that in SK‐216 treated group. On the other hand, the expression of the epithelial marker, e‐cadherin was not changed (Figure 4F).

### 3.6 The level of PAI‐1 expression correlated with α‐SMA expression of CAFs and was associated with lung cancer progression

In this study, we investigated the association between PAI‐1 and chemotherapy resistance. Finally, to investigate whether PAI‐1 expression correlated with the MF characteristic was associated with lung cancer progression, we examined the correlation between the PAI‐1 expression in lung cancer tissue and T, N factor and stage. We showed that PAI‐1 expression was significantly higher in the patients with T 3/4, stage 2/3 than in the patients with T 1/2, stage 1 respectively (Table 2).

**Table 2:** The correlation between PAI‐1, α‐SMA expression and patient characteristics

Patient characteristics	No.	PAI‐1 (%)	*P* value	α‐SMA (%)	*P* value
		Mean ± SD		Mean ± SD	
Age					
≦65	18	2.6 ± 0.6	NS	6.5 ± 1.1	NS
>65	16	5.0 ± 0.9		5.9 ± 1.4	
Sex					
Male	27	4.3 ± 0.7	NS	6.6 ± 1.0	NS
Female	7	2.6 ± 1.1		4.8 ± 1.6	
T factor					
1, 2	28	3.2 ± 0.6	0.007	5.6 ± 1.0	NS
3, 4	6	7.1 ± 1.1		7.1 ± 1.1	
N factor					
0	20	3.0 ± 0.7	NS	4.4 ± 1.1	NS
1‐3	14	5.2 ± 1.0		6.0 ± 1.2	
Stage					
I	16	2.0 ± 0.5	0.002	2.9 ± 1.1	<0.001
II, III	18	5.7 ± 0.8		9.1 ± 0.9	

NS, not significant.

## 4 DISCUSSION

In this study, we showed that the level of PAI‐1 was correlated with the proportion of MF characteristics of CAFs in lung adenocarcinoma. Further, we revealed that PAI‐1 was associated with maintaining the MF characteristics of fibroblasts via inhibition of apoptosis. Furthermore, the inhibition of PAI‐1 suppressed the MF characteristics of fibroblasts and this suppression increased the effect of chemotherapy on lung cancer cells. Finally, we showed that the level of PAI‐1 in the lung adenocarcinoma was also associated with the tumour progression.

A large number of studies showed that cancer stromal cells, such as inflammatory cells, endothelial cells and fibroblasts, induced resistance to chemotherapy in lung cancer.^25^ In the stromal cells, fibroblasts have been reported to be a main player in this phenomenon.^4^, ^26^ In the present study, we revealed that PAI‐1 inhibition limited the chemotherapy resistance through suppressing the MF characteristics of CAFs. In addition, we showed that the level of PAI‐1 produced by CAFs was higher than that produced after suppression of the MF characteristics of CAFs by PAI‐1 inhibition. This result indicates that there could be a positive feedback loop between PAI‐1 and the MF characteristics of CAFs (Figure S5). Thus, PAI‐1 could be a reasonable therapeutic target to inhibit resistance to chemotherapy in the lung cancer with CAFs.

In this study, PAI‐1 maintained the MF characteristics of CAFs through inhibiting apoptosis. PAI‐1 is reported to be involved in various cancer cell properties such as migration, invasion and proliferation.^8^, ^27^, ^28^, ^29^ Several studies showed PAI‐1 was involved in these processes through association with apoptosis,^18^ PI3k/Akt, MAPK^17^, ^30^ and TGF‐β signalling including EMT.^10^, ^24^ In this study, when CAFs were treated with a PAI‐1 inhibitor, the greater number of proteins related to apoptosis were up‐regulated as compared to that of MAPK or TGF‐β signalling proteins as observed by quantitative proteomic analysis. On the other hand, the degree of phosphorylation of MAPK protein was not different between CAFs treated with SK‐216 and untreated control (Figure S4A and B). Furthermore, we previously revealed that PAI‐1 inhibition in fibroblasts was not associated with TGF‐β inducing Smad signalling pathway and non‐Smad signalling pathways, including ERKs.^10^ PAI‐1 was also shown to be involved in the apoptosis of cancer cells,^18^ endothelial cells, and neurons.^31^ From these observations, we conclude that apoptosis is an important mechanism by which PAI‐1 maintains the MF characteristics of CAFs.

Several reports showed that CAFs promote tumour progression and resistance to chemotherapy by secreting various growth factors and inflammatory chemokines.^4^, ^5^ In this study, the level of PAI‐1 and TGF‐β secreted after suppression of the MF characteristics of CAFs by PAI‐1 inhibition was significantly lower than that from CAFs without PAI‐1 inhibition. Thus, we considered the PAI‐1 and TGF‐β secreted by CAFs could be associated with resistance to chemotherapy in cancer cells in this study. PAI‐1 and TGF‐β were reported to be associated with EMT of cancer cells in the absence of CAFs.^10^ In this study, the expression of mesenchymal genes was elevated in the cancer cells co‐cultured with CAFs compared with CAFs treated with PAI‐1 inhibitor. This result suggested that EMT induced by PAI‐1 and TGF‐β in cancer cells was associated with decreasing the effect of chemotherapy in this study, as previously described.^10^, ^17^, ^18^

Previous studies revealed that PAI‐1 produced by cancer cells was associated with cancer progression and prognosis in various malignancies.^32^, ^33^, ^34^ On the other hand, we previously reported that host, but not tumour, PAI‐1 is important in lung cancer progression in vitro and in vivo.^15^ In this present study, we showed that the high level of host PAI‐1 expression in the tumour stroma was associated with tumour size and the TNM stage using immunohistochemical analysis of lung cancer tissue. Our findings suggest that PAI‐1 could be a suitable treatment target for progressive tumour with abundant stroma cells that produce PAI‐1.

Our data suggest that PAI‐1 may influence the tumour progression and effectiveness of chemotherapy through its association with the MF characteristics of CAFs. Hence, PAI‐1 might become a promising target for anti‐tumour therapy in the lung cancer with CAFs.

## AUTHORS’ CONTRIBUTIONS

TM, NH contributed to study conception and design; TM, TN, MN, KY, SS, YH, SM, IH, KF, YM, HH, MO, NH performed acquisition of data; TM, TN, NM, NH contributed to analysis and interpretation of data; TM, TN contributed to the drafting of manuscript. All authors read and approved the final manuscript.

## CONFLICTS OF INTEREST

The authors confirm that there are no conflicts of interest.
