# Supplementary figures and images for: Inhibition of PAI‐1 limits chemotherapy resistance in lung cancer through suppressing myofibroblast characteristics of cancer‐associated fibroblasts
Source: J Cell Mol Med. 2019 Feb 7;23(4):2984–94. doi: 10.1111/jcmm.14205 (PMC6433668; doi:10.1111/jcmm.14205)

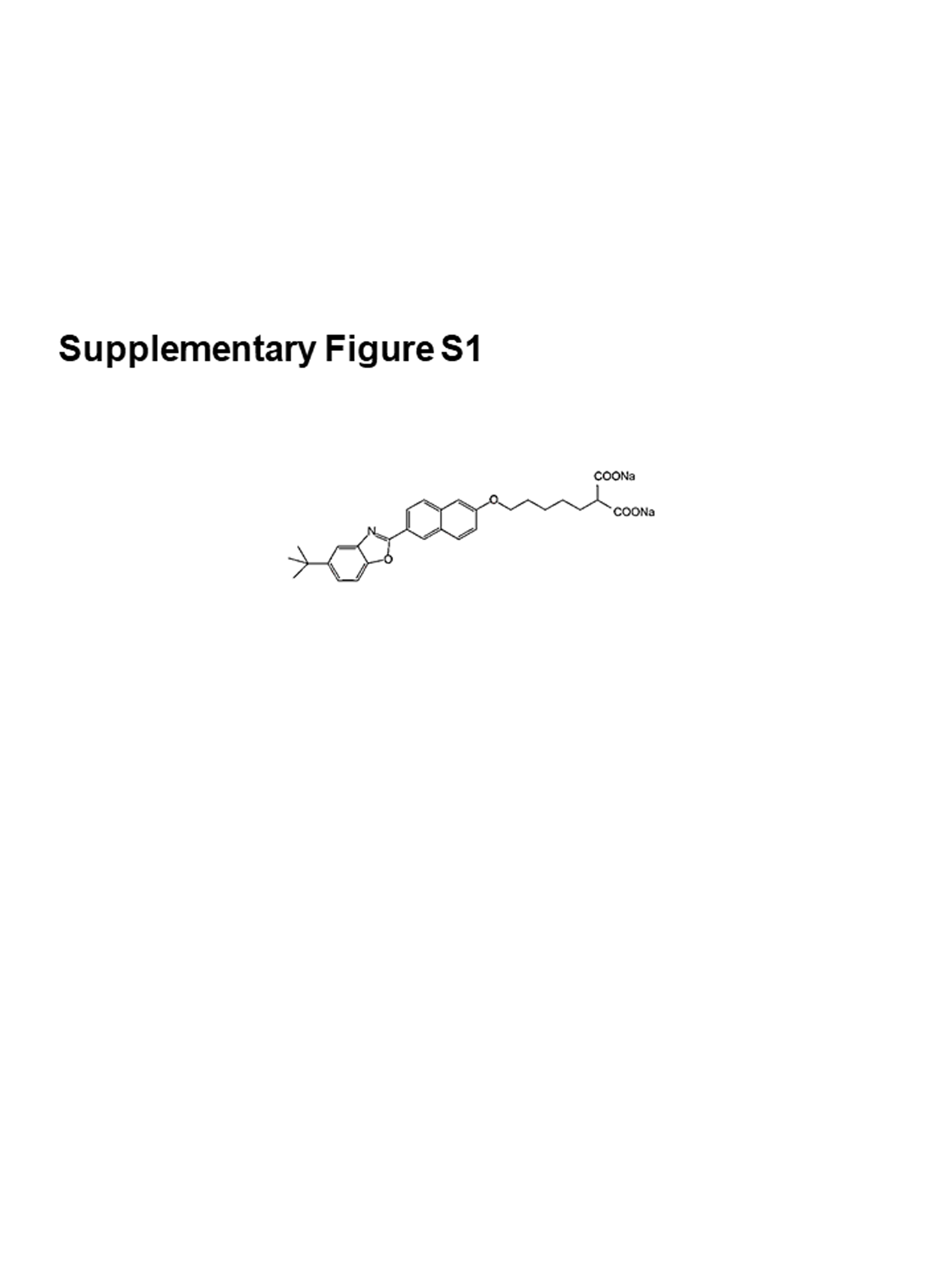

Supplement: Supplementary file 1 [file JCMM-23-2984-s001.tif]

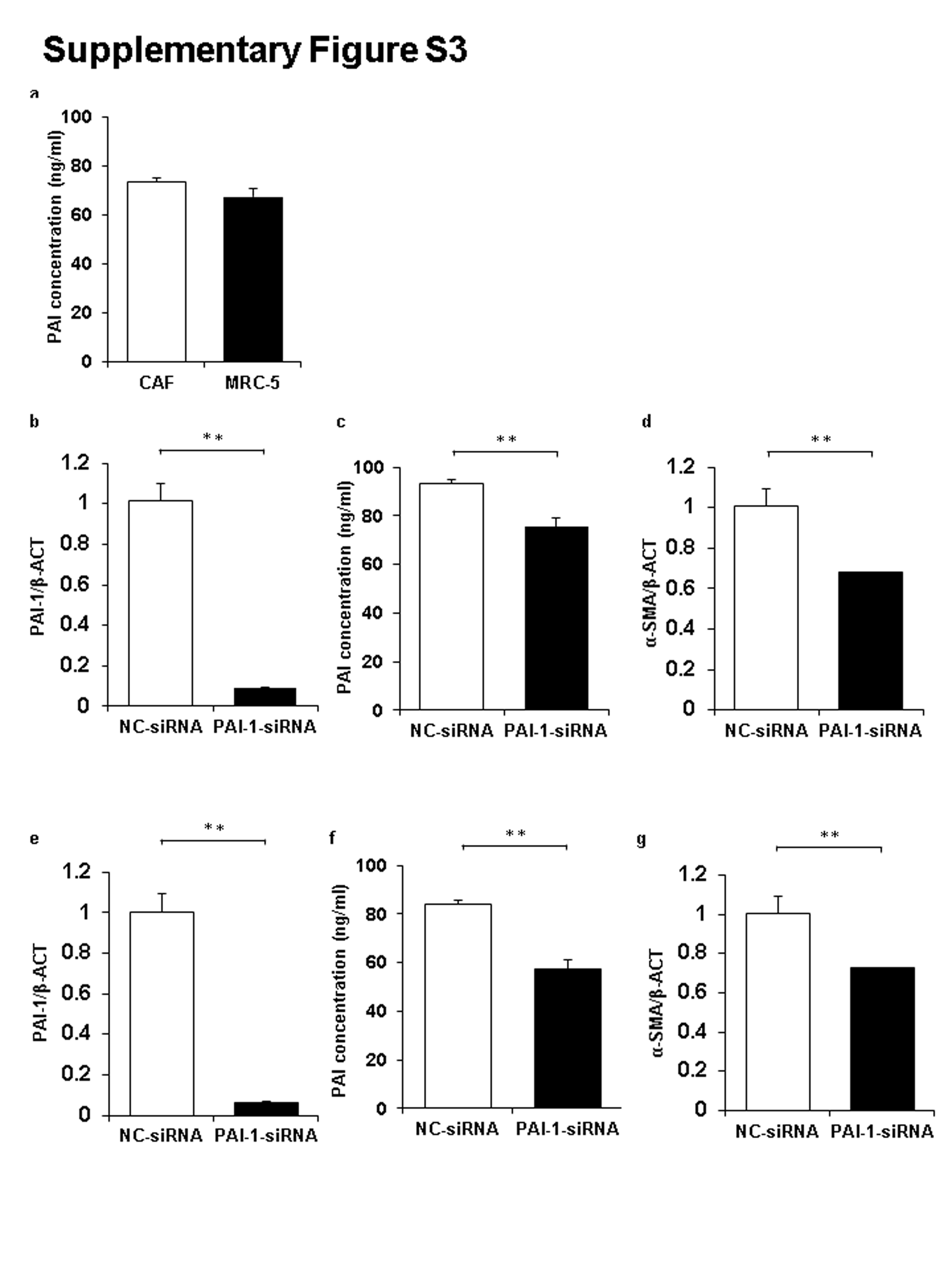

Supplement: Supplementary file 3 [file JCMM-23-2984-s003.tif]

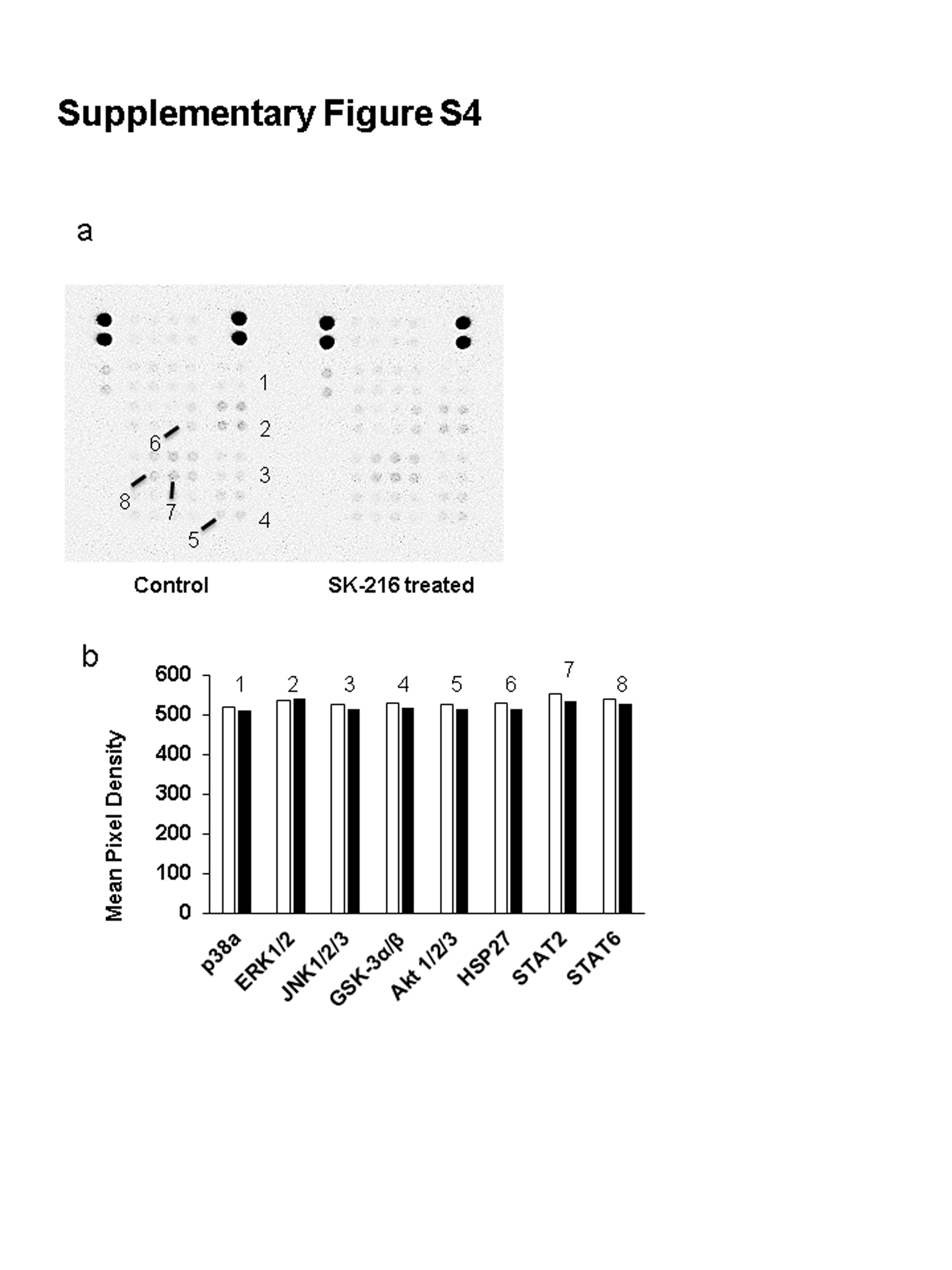

Supplement: Supplementary file 4 [file JCMM-23-2984-s004.tif]

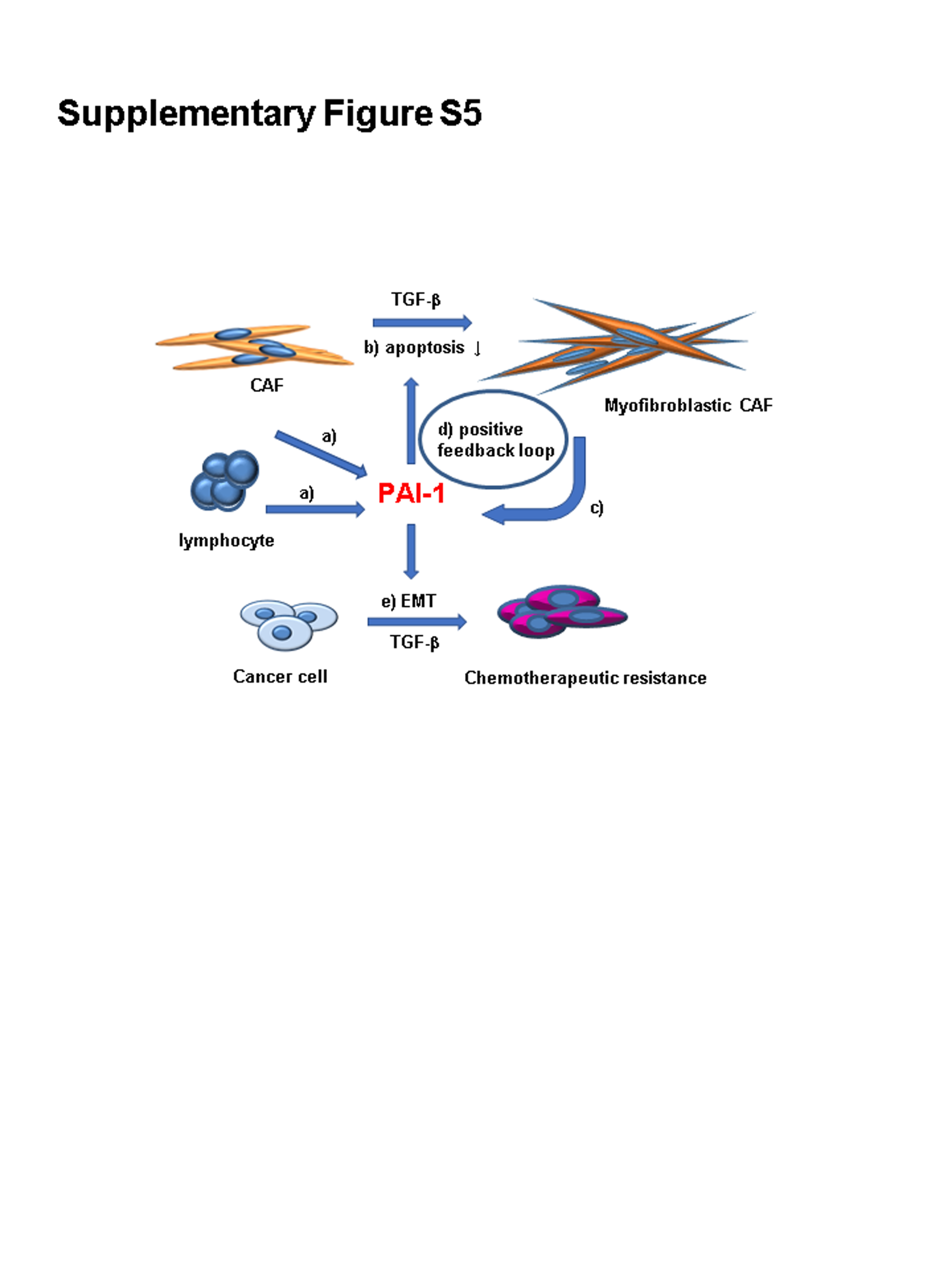

Supplement: Supplementary file 5 [file JCMM-23-2984-s005.tif]
