# Supplementary material for: Inhibition of PAI‐1 limits chemotherapy resistance in lung cancer through suppressing myofibroblast characteristics of cancer‐associated fibroblasts
Source: J Cell Mol Med. 2019 Feb 7;23(4):2984–94. doi: 10.1111/jcmm.14205 (PMC6433668; doi:10.1111/jcmm.14205)
Supplement: Supplementary file 6 [file JCMM-23-2984-s006.docx]

Supplementary Table 1

16-upregulated protein related to apoptosis of CAF after PAI-1 inhibitor treatment

| Protein name | ｆold change | p-value |
| --- | --- | --- |
| Prelamin | 2.2379 | 0.0009 |
| Apoptosis inhibitor 5 | 1.8562 | 0.0208 |
| Translocation protein SEC62 | 1.8305 | 0.0182 |
| Matrin-3 | 1.7286 | 0.0342 |
| PRP4 pre-mRNA processing factor 4 | 1.6469 | 0.0108 |
| Nucleolin | 1.6152 | 0.0209 |
| Thyroid hormone receptor-associated protein 3 | 1.5952 | 0.0361 |
| Chromobox homolog 3 | 1.52 | 0.0235 |
| Bcl-2-associated transcription factor 1 | 1.4897 | 0.0008 |
| Epididymis luminal protein 35 | 1.4638 | 0.0271 |
| Trans-Golgi network integral membrane protein 2 | 1.4082 | 0.0399 |
| Isoform 2 of Calnexin | 1.4052 | 0.0126 |
| Protein IWS1 homolog | 1.3936 | 0.0077 |
| Periphilin-1 | 1.3461 | 0.0308 |
| Nuclease-sensitive element-binding protein 1 | 1.249 | 0.0445 |
| Epididymis luminal protein 113 | 1.1782 | 0.0198 |
